# Design and Validation of a Diet Rich in Slowly Digestible Starch for Type 2 Diabetic Patients for Significant Improvement in Glycemic Profile

**DOI:** 10.3390/nu12082404

**Published:** 2020-08-11

**Authors:** Aurélie Goux, Anne-Esther Breyton, Alexandra Meynier, Stéphanie Lambert-Porcheron, Monique Sothier, Laurie Van Den Berghe, Olivier Brack, Sylvie Normand, Emmanuel Disse, Martine Laville, Julie-Anne Nazare, Sophie Vinoy

**Affiliations:** 1 Nutrition Research, Mondelēz International, 91400 Saclay, France; aurelie.goux@mdlz.com (A.G.); Anne.Breyton@mdlz.com (A.-E.B.); alexandra.meynier@mdlz.com (A.M.); 2 Centre de Recherche en Nutrition Humaine Rhône-Alpes, Univ-Lyon, CarMeN Laboratory, Université Claude Bernard Lyon1, Hospices Civils de Lyon, F-CRIN/FORCE Network, 69100 Pierre Bénite, France; stephanie.lambert-porcheron@chu-lyon.fr (S.L.-P.); moniquesothier@wanadoo.fr (M.S.); ext-laurie.vandenberghe@chu-lyon.fr (L.V.D.B.); sylvie.normand@chu-lyon.fr (S.N.); emmanuel.disse@gmail.com (E.D.); martine.laville@univ-lyon1.fr (M.L.); julie-anne.nazare@univ-lyon1.fr (J.-A.N.); 3 Service Endocrinologie-Diabète-Nutrition, Hospices Civils de Lyon, 69002 Pierre-Bénite, France; 4 Statistique Industrielle KHI2 Consulting (KSIC), 60110 Esches, France; olivier.brack@wanadoo.fr

**Keywords:** slowly digestible starch, diet, type 2 diabetes, glycemic response, continuous glucose monitoring system

## Abstract

This study aimed at designing a—diet high in slowly digestible starch (SDS) by carefully selecting high-SDS starchy products and to validate its implementation, acceptance, and impact on the postprandial glycemic response in patients with type 2 diabetes (T2D). Starchy products were screened and classified as being either high (high-SDS) or low (low-SDS) in SDS (in vitro SDS method). A randomized controlled cross-over pilot study was performed: Eight patients with T2D consumed randomly a high-SDS or a low-SDS diet for one week each, while their glycemic profile was monitored for 6 days. Based on 250 food product SDS analyses and dietary recommendations for patients with T2D, the high-SDS and low-SDS diets were designed. The high-SDS diet significantly increased SDS intake and the SDS/carbohydrates proportion compared to the low-SDS diet (61.6 vs. 11.6 g/day and 30% vs. 6%; *p* < 0.0001, respectively). Increasing the SDS/carbohydrate proportion to 50% of the meal was significantly correlated with a 12% decrease in tAUC0–120 min and a 14% decrease in the glycemic peak value (*p* < 0.001 for both). A high-SDS diet can be easily designed by carefully selecting commercial starchy products and providing relevant recommendations for T2D to improve their glycemic profile.

## 1. Introduction

According to recent data from the International Diabetes Foundation, diabetes is a major health concern, and its incidence is expected to increase by 51% by 2045 [1]. Recent scientific consensus states that the treatment goals for type 2 diabetes (T2D) are to prevent or delay complications and to maintain quality of life. Managing daylong glycemia in adults with T2D is a key factor in attaining those goals [2,3]. Lifestyle interventions are an effective and safe means of improving glucose control and should be part of the treatment strategy for all patients with T2D. This approach includes nutritional advices (focusing especially on dietary quality and energy restriction), physical activity, weight loss, counseling for smoking cessation, and psychological support [3,4,5]. Regarding diet quality, carbohydrates account for the largest part of the diet, ranging from 45% to 60% for patients with T2D [4,6,7]. In addition, it has been demonstrated that carbohydrate quality (mainly based on a low glycemic index (GI) approach), beyond quantity, is key in managing the cardiometabolic risks associated with diabetes [8,9]. Starch-based products can play a role in the prevention of hyperglycemic events. More specifically, starch was classified into three fractions based on its digestibility rate by Englyst and collaborators: Rapidly digestible starch (RDS), slowly digestible starch (SDS), and resistant starch (RS) [10,11]. The SDS content and food matrix composition in fat and fibers are strongly influencing the postprandial physiological responses (glycemic index and glycemic responses) in cereal products [12]. Various studies have demonstrated that a high SDS content is a key factor in decreasing postprandial glycemic and insulinemic responses in healthy adults [13,14,15,16,17] and in insulin-resistant subjects [18] on single eating occasions. Longer term studies that investigated the effect of increasing the SDS content as a way to modulate a low-GI diet demonstrated reduced postprandial glycemia and insulinemia and improved cardiovascular risk factors in healthy overweight adults [14,19]. The beneficial effect of slowing the rate of starch digestion holds promise for treating T2D, although up to day, it has mainly been investigated using raw starch [20,21,22,23] as nobody investigated the SDS content of a wide range of commercially available products.

The aim of this study was to design a diet with a high SDS content by carefully selecting high-SDS starchy food products and to validate its implementation, acceptance, and impact on the postprandial glycemic response in patients with T2D.

## 2. Materials and Methods

### 2.1. High-SDS Diet and Low-SDS Diet Design

The starch digestibility profile, including the SDS content, of a wide range of commercially available starchy products was measured using the SDS method [11,24]. SDS content analyses were carried for all food groups rich in starch content that are representative of a standard French diet for T2D: Rice, pasta, other wheat products, other cereal products, legumes, potatoes, bread/bread substitutes, and biscuits. More than 250 starch digestibility analyses of ready-to-eat or cooked products were carried out. Within each food group, SDS content analyses were performed for a variety of products (various origins; different product shapes for food, such as pasta; traditional vs. quick-cooking products, etc.), from various brands with a range of cooking instructions (suppliers’ vs. alternate cooking recommendations).

The diet was designed to provide at least one serving of a starch-based product per meal and to comply with dietary recommendations for patients with T2D [6,7]. Trained dieticians provided individualized dietary advice to patients regarding their usual dietary intake (both quantitative and qualitative) to ensure a steady carbohydrates and starch intakes and an adequate SDS intake. Specific cooking instructions with menu examples, including the recommended starch-based products, were provided to the patients. Among the products included in the diet, the SDS content of 53 selected commercial products was monitored over the course of the study to ensure that there were no major changes in SDS content during the experimental phase.

### 2.2. Study Design

A single-blind monocentric randomized controlled cross-over pilot study was designed to evaluate the feasibility of consuming the high-SDS and low-SDS diets and to test their postprandial glycemic impacts. The study protocol was approved by the local ethics committee (reference number 2017-A01160-53, Sud-Est I) and was registered with clinicaltrials.gov (NCT 03289494). Patients randomly consumed for one week (7 days +/− 1 day), a high-SDS diet and a low-SDS diet with a two-week +/− 3-day wash-out period. To ensure that only the starch quality of the diets differed, the patients were required to consume the same menus during both weeks, except for the starch-based products being selected from either the high-SDS or low-SDS diet.

At the beginning of each nutritional intervention week, patients came to the research center (Centre de Recherche en Nutrition Humaine Rhône-Alpes) for their inclusion visit, to have a medical interview and their anthropometric and vital sign data recorded. After randomization, the patients met with trained dieticians who instructed them on how to follow the diet recommendations during the upcoming week. The starch-based products for their first allocated diet, along with the relevant cooking instructions, were provided to the patients. A continuous glucose monitoring system (CGMS) device was inserted into patients’ upper arms to monitor their glycemic profiles during each dietary intervention week. Patients were instructed to continue their usual daily routine and to complete a daily dietary diary during each nutritional intervention week.

At the end of each nutritional intervention week, the patients returned to the research center, in a nonfasted state, to have their anthropometric and vital signs measured and to return any unconsumed starchy products. The CGMS device was removed, and the recorded data were downloaded.

### 2.3. Diet Composition, Compliance, and Acceptance

Dieticians analyzed the seven-day dietary diaries from both nutritional intervention periods to assess compliance with the diet and to determine the macronutrient and SDS contents of the diets (using Nutrilog^®^ software, version 3.10b, released in February 2017). SDS contents of the diets were calculated using the analytical results obtained during the design of the diets. Dieticians also estimated compliance with the diets based on patients’ statements during the medical interviews and on the amount of unconsumed starch-based products that was returned.

Patients filled in feedback questionnaires at the end of both dietary intervention periods to evaluate the feasibility and acceptability of each diet over a one-week period and the expected tolerance over longer periods.

### 2.4. Population

Eight patients with T2D (2 men and 6 women) finished the study. The main inclusion criteria were: age 18–75 years old, BMI 22–37 kg/m^2^, stable weight for at least 3 months, HbA1c 6.5–8.5%, treatment with metformin and sitagliptin (DPP-IV inhibitors) at a stable dose for at least 1 month, triglycerides < 4 g/L, Low Density Lipoprotein-cholesterol < 1.90 g/L, C-reactive protein < 15 mg/L, non-smokers, moderate alcohol consumption (<20 g alcohol/day), not on a restrictive or specific diet, regular consumption of three 3 main meals per day, and stable and moderate physical activity (<4 h/week).

### 2.5. Postprandial Glycemic Response

Glycemia was measured using the Medtronic iPro^®^2 CGMS device (Medtronic Minimed, Northridge, CA, USA). Mean interstitial fluid glucose values were recorded every 5 min (for a total of 288 reads per day) for up to 6 consecutive days. Patients were not able to access the readings. At the end of each dietary intervention period, the CGMS data were downloaded using Medtronic “CareLinkPro” software. Capillary blood glucose was also measured for calibration purposes using finger-stick blood samples at least four times a day using a blood glucometer Contour^®^XT (ASCENSIA, Diabetes Care, Basel, Switzerland), and the values were recorded in a blood glucose diary.

Seven patients were included in the final CGMS data analysis due to recording failure during both holding periods for one patient. The complete datasets for the seven included patients captured data from lunch on day 1 to dinner on day 6, representing a total of 237 meals (missing data for one meal), and these data were used to determine postprandial glycemic parameters and associated macronutrient and SDS content consumptions.

From the CGMS raw data, calculated postprandial glycemic parameters included the total area under the curve (tAUC) and time in range (TIR, %), both of which were calculated for a 120-min interval after each meal and a 240-min interval after lunch and dinner. The 240-min interval breakfast calculations were impossible because lunch was often consumed before the end of the 4-h postprandial period. TIR represents the time spent by each patient in one of five specific glycemic ranges: <70 mg/dL, [70–140 mg/dL[, [140–180 mg/dL[, [180–250 mg/dL], and >250 mg/dL. The glycemic peak value, delta peak value, and time to reach the peak value were also calculated. A detailed description of these parameters is available elsewhere [25].

### 2.6. Statistical Analyses

A standard power calculation for this pilot study was not possible due to the lack of available data on low-SDS and high-SDS diets and their impact on a T2D population. Based on the literature evaluating postprandial glycemic responses in healthy subjects with products containing SDS, we decided to include eight patients, as one clinical trial that included only seven subjects demonstrated a significant difference in glycemic response [26].

Data are reported as mean ± standard error of the mean (SEM). Differences between paired data for the high-SDS and low-SDS diets in terms of energy, macronutrient composition, fibers, and SDS content were assessed using Student’s t-test for normally distributed data, or Wilcoxon test for data with a non-normal distribution.

To evaluate the correlation between postprandial glycemic parameters and the proportion of SDS among available carbohydrates (SDS/carbohydrates) in the diet, a quadratic model was first built, followed by a linear model if no quadratic effects were reported. The following effects were included in the model: SDS/carbohydrates, (SDS/carbohydrates)^2^ (quadratic model only) and subject as a random effect.

Statistical analyses were performed using JMP 14.0^®^(SAS-Institute, Cary, NC, USA). *p* values < 0.05 were considered significant.

## 3. Results

### 3.1. SDS Analysis of Commercial Food Products and Diet Design for the Study

The starchy component of the high-SDS and low-SDS diets was designed to maximize the difference in SDS content between the two diets. Initially, we set a minimum SDS content threshold of 15 g/100 g as a target for foods to be included in the high-SDS diet. However, only biscuits, rice, and pastas met this target, so for the remaining food groups (other wheat products, other cereal products, legumes, potatoes, bread/bread substitutes), we selected products with an SDS content at either extreme of the range identified for that food group to be included in the high-SDS or low-SDS diet. One or more starchy product alternatives per food group were selected to promote consumption of a diverse ranges of foods and to ensure adequate compliance to the diet. Recommendations for the frequency of consumption were also provided based on the SDS content of the products. More than 250 SDS content analyses were performed (70 analysis for rice, 57 for pasta, 18 for other wheat products, 5 for other cereal products, 16 for legumes, 12 for potatoes, 44 for bread/bread substitutes, and 48 for biscuits), and 136 SDS content analyses of the tested food products were included in the diet design. The range of SDS contents of the selected products for each food group are shown in Table 1, which demonstrates the actual differences in SDS content between the two diets. More detail regarding both diets (products included, cooking instructions, SDS and SDS/carbohydrates values, and recommended frequency of consumption) can be found in Tables S1 and S2. Overall, quick-cooking products seemed to have lower SDS contents than their traditional counterparts, and canning decreased the SDS content of legumes. Notably, no cereal products other than wheat-based products and rice were found to have high SDS contents. It is also important to highlight that increasing the cooking time diminished the SDS content. A slight decrease in the SDS content was observed for each additional two minutes of cooking time, but cooking conditions were strictly regulated in this study to maintain the highest SDS levels.

Throughout the study, the SDS content of 53 commercial products was monitored, and for the most part, no major variations in SDS content were detected between the start and the end of the experimental phase. The analytical difference in the SDS content between batches of the same product was below the method variability (2 g/100 g) [11]. One type of pasta included in the low-SDS diet demonstrated a 5 g/100 g increase in the SDS content at the end of the study period compared with the beginning. A bread substitute and a pasta included in the high-SDS diet exhibited slightly lower SDS values at the end of the study period compared with the beginning, near the limit of detection for method variability (2 g/100 g).

### 3.2. Study Population Characteristics

Eight patients with T2D (male/female: 2/6) finished the study, all of who were taking metformin and sitagliptin. The mean patient age was 59.8 ± 3.0 years old. All the patients were overweight or obese (mean BMI = 31.7 ± 2.1 kg/m^2^), with HbA1c between 6% and 8% (mean = 7.0 ± 0.2%), mean fasting glycemia of 7.4 ± 0.4 mM, and a mean duration of diabetes of 10 ± 2 years. Body weight and BMI did not significantly differ between the first and the last visit.

### 3.3. Diets Composition

The macronutrient composition over a period of one week was equivalent for both diets when expressed as the percent of total daily energy intake (TDEI), except for fiber consumption, which was slightly but significantly lower (0.3% lower) for the low-SDS diet compared with the high-SDS diet (*p* < 0.05) (Table 2). Patients consumed more calories (*p* < 0.05) on the high-SDS diet compared to the low-SDS diet, due to a small higher intake in carbohydrate (*p* < 0.001), also accompanied with a small but significant increase in fiber consumption (*p* < 0.01) (Table 2).

Consistent with the aim of the diet, daily SDS consumption on the high-SDS diet was significantly higher (by 5-fold) than on the low-SDS diet. This increase in SDS consumption was significant on a per-day basis and for each individual meal throughout the day (*p* < 0.0001 for the day, breakfast, lunch, and dinner) (Table 3). The SDS/carbohydrate ratio (which was assessed to account for the moderate increase in carbohydrate consumption in the high-SDS diet group) was also significantly higher for the high-SDS diet compared to the low-SDS diet for the day and for each meal (*p* < 0.0001 for the day, breakfast, lunch, and dinner). SDS accounted for 27–32% of the available carbohydrates in the high-SDS diet compared to 3–10% in the low-SDS diet (Table 3).

### 3.4. Contribution of Various Food Groups to the SDS Intake for Both Diets

The SDS intake over 6 days demonstrated that pasta and rice were the most important contributors to SDS intake for both diets. For the high-SDS diet, three product categories accounted for up to 80% of the SDS intake: Pasta, rice, and biscuits (Table 4). This is consistent with the SDS contents of these three groups of products being in the highest SDS range (Table 1).

### 3.5. Compliance to the Diet Interventions

Thirty-six meals per patient (breakfast, lunch, and dinner) were consumed over the two weeks of dietary intervention (high-SDS and low-SDS diets), representing a total of 288 meals for the entire study. Overall, the compliance with both diets was very good. Only 8 out of the 288 meals (2.8%) showed issue in food consumption frequency recommendations (five meals did not contain any starch-based food; overconsumption of low-GI bread in three meals). Furthermore, 100% of the cooking instructions were respected. Finally, the analysis of the satisfaction questionnaires demonstrated that patients appreciated the dietary program and overall found it easy to follow. No significant difference was found in the satisfaction with each diet (*p* > 0.05).

### 3.6. Correlation between the SDS/Carbohydrates Ratio for a Meal and the Postprandial Glycemic Responses

**Figure 1:**
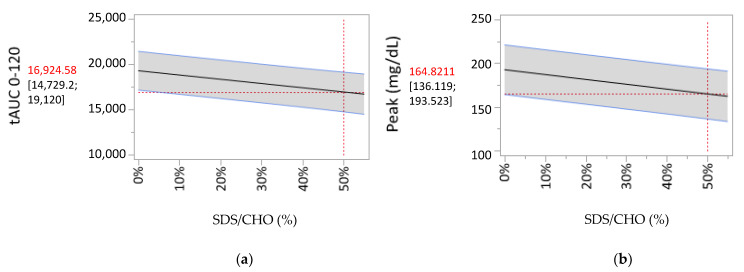
Prediction profiler representation of the linear correlations between the SDS/carbohydrate content of meals and (**a**) tAUC0–120 min and (**b**) the peak glycemic value. The grey zone represents the 95% confidence interval.

To account for the slight increase in carbohydrate intake in the high-SDS diet compared to the low-SDS diet, the SDS/carbohydrate ratio was investigated to evaluate the impact of increasing the SDS content of meals. Increasing the SDS/carbohydrate ratio of a meal induced a significant decrease in tAUC0–120 min, peak value, delta peak value, and the time spent in the two highest glycemic range for the 2-h postprandial period (Table 5). For instance, increasing the SDS/carbohydrate ratio of the meal to 50% led to a 12% decrease in tAUC0–120 min and a 14% decrease in the peak glycemic value (Figure 1). Additionally, a 30% SDS/carbohydrate ratio, representing the mean content of the high-SDS diet, induced a 7% and 8% decrease in tAUC0–120 min and the peak glycemic value, respectively. The time spent in the highest glycemic range (TIR > 250 mg/dL) during the 2-h postprandial period demonstrated a significant quadratic interaction. This means that increasing the SDS/carbohydrate ratio of a meal decreased the amount of time during which patients exhibited glycemic values above 250 mg/dL in a nonlinear fashion; below 20% of SDS/carbohydrate in a meal, the reduction is the strongest, from 20% to 30% of SDS/carbohydrate, the decrease is less pronounced, and above 30% of SDS/carbohydrate ratio in a meal, no additional effect can be observed as no values are above 250 mg/dL (Table 6).

Only lunch and dinner were analyzed in terms of the 4-h postprandial periods, as the time that elapsed between breakfast and lunch was usually not long enough to calculate relevant parameters. Increasing the SDS/carbohydrate ratio in a meal increased significantly in a linear fashion the time spent in the glycemic range of 140 to 180 mg/dL during the 4-h postprandial. This was paralleled by a trend toward decreased time spent at the two highest glycemic ranges (>180 mg/dL) during the 4-h postprandial period (Table 5 and Table 6). The detailed results about the glycemic parameters can be found in in one recent manuscript (in press) in which all CGMS data were fully described [25].

## 4. Discussion

The aim of this pilot study was to design a high-SDS diet and validate it by showing the benefit of increasing the dietary SDS content on glycemic control for T2D management. To achieve this goal, we used a step-by-step approach. First, we designed two diets containing significant different levels of SDS that both respected T2D dietary recommendations and the cultural habits of the country where the study took place [6,7]. Second, after one week’s consumption of the high-SDS diet, we confirmed that there was a significantly higher SDS intake and demonstrated that consuming a high-SDS diet was appreciated and easily implementable by patients. Finally, this pilot study demonstrated an improvement in postprandial glycemic response parameters when patients with T2D taking metformin and sitagliptin consumed meals with a higher SDS/carbohydrate ratio for one week.

As part of the present study, we determined the SDS content of a wide range of commercially available starchy foods. Carefully selecting these products for a high SDS content, considering industrial and home cooking preparation steps, may be beneficial in terms of managing the postprandial glycemic response in patients with T2D.

SDS analysis showed that the SDS content of legumes was in the lowest range. It has been previously reported that commercial canning increases the in vitro starch digestibility of beans and the in vivo postprandial metabolic responses, although these latter were still relatively low [27]. We based the inclusion of legumes in our diet design on this observation, and included either canned or boiled legumes in the low-SDS diet or high-SDS diet, respectively. Patients with T2D are advised to consume legumes at least once a week, and these results could help to finetune recommendations on legumes. We did not identify potatoes and bread with a high-SDS content. Exclusion of these two products, which are regularly consumed as part of a typical French diet, could have led to compliance issues. We included two products identified from the literature in the high-SDS diet under the following conditions: (1) Controlled frequency of consumption, and (2) the inclusion of low-GI products to comply with the main aim of this diet [28,29].

The goal of nutritional recommendations provided to patients with T2D is to improve glycemic control and manage cardiovascular risk factors, but little specific advice on carbohydrates was provided as part of the latest global recommendations [3]. Some earlier recommendations considered the consumption of carbohydrate-rich low-GI foods to be appropriate [6,30]. The consideration of GI was very recently included in the French recommendations for T2D, which explain that variations in GI may be linked to the degree of starch gelatinization in starchy products [31]. An example was provided regarding the preparation of pasta, which is recommended to be eaten al dente. The SDS content is strongly related to the level of starch gelatinization [32] and to the GI of starchy foods [12]. The present study provides much more extensive information regarding the preparation of foods from several food groups and their SDS content. Indeed, the results from this study provide a strong rationale for the recommendations linked to starch gelatinization and suggest that SDS content is a simple and measurable in vitro parameter that reflects the CHO quality of the diet.

This dietary intervention had multiple strengths. Providing cooking instructions along with dietary recommendations, advice on consumption frequency, and a supply of starchy foods made the diet easy to implement and was appreciated by patients in a real-life setting. Dietary diaries allowed us to monitor the macronutrient and SDS intake at each meal for both diets. The total energy intake was higher for the high-SDS diet compared with the low-SDS diet, due to a slightly higher intake of available carbohydrate. However, the proportions of macronutrients consumed for both diet interventions remained constant. The estimated total energy intake for both diets seemed quite low and may have been under-reported by patients, as it has been demonstrated previously [33]. The overall prevalence of energy intake under-reporting in French adults is 22.5%, and is positively associated with overweight among other factors [33]. For this proof-of-concept pilot study, we included products in all food groups. However, removing these two products would have led to greater differences in SDS intakes and potentially in glycemic response too. Additionally, as the products included in the high-SDS diet are commercial products, their consumption is restricted by their availability from the various suppliers and the potential for manufacturing processes to change during the life of the products. We checked the latter point by measuring the SDS levels of the commercial products consumed as part of this study to ensure that no major SDS content changes occurred during the study period.

Correlations between SDS/carbohydrate ratios and the postprandial glycemic responses were investigated to validate the impact of increasing the SDS content of meals. There was a significant inverse correlation between the SDS/carbohydrate ratio and peak glycemic value, time spent in higher glycemic ranges (TIR > 180 mg/dL), and tAUC during the 2-h postprandial period. This demonstrates that as the SDS content of the meal increases, the postprandial glycemic response in patients with T2D significantly decreases, especially in the first two hours postprandially. Modulating the amplitude of the increase in SDS content thus appears to be a useful approach for improving postprandial glycemia. These results are consistent with previous studies that reported a significant decrease in the glycemia iAUC in patients with T2D after consumption of slowly hydrolyzed starch [20] or a significant decrease in the mean daily glycemic demand after consumption of a slow-starch product at breakfast for 2 weeks [23]. As the SDS content is key in explaining the GI of the products, these results are promising, given that two meta-analyses reported a significant decrease in mean HbA1c in diabetic patients when consuming low-GI diets, by 0.43% for shorter term studies (10 weeks’ mean duration) and by 0.14% for studies lasting longer than 6 months [30,34]. Two recent meta-analyses are key to explaining the mechanism of action associated with SDS. First, a high SDS content is a key factor for decreasing the rate of appearance of exogenous glucose (RaE) in the blood stream, and thus for reducing the glycemic and insulinemic responses in healthy adults. This study showed a 15-fold greater chance of having a low RaE and a 5-fold greater chance of having a low glycemic response after consuming a high-SDS food product compared to a low-SDS food product [35]. In addition, a reduction in the RaE was associated with a significant reduction in the postprandial glycemic and insulinemic responses [36]. The present work demonstrates that in patients with T2D, consuming a diet with a high SDS content may be a valuable approach to lowering the postprandial glycemic response. We hypothesize that this effect is mediated by a decrease in RaE, which generates a better glycemic profile with lower postprandial responses and is potentially associated with a lower insulin demand, leading to beneficial metabolic effects. The current study shows that a higher SDS content correlates with an improvement in glycemic profile parameters, such as peak values, postprandial tAUC, and even TIR results, meaning that patients who consume more SDS achieve more appropriate postprandial glycemic targets. Additional results from this study based on analysis of the CGMS data demonstrate significantly lower glycemic variability following the high-SDS diet compared to the low-SDS diet for the mean amplitude of glycemic excursions (MAGE; *p* < 0.01), standard deviation (SD; *p* < 0.05), and coefficient of variation (CV; *p* < 0.01) [25].

## 5. Conclusions

A high-SDS diet can be easily designed by carefully selecting commercially available starchy products and providing relevant recommendations for T2D. For the first time, we showed that controlling starch digestibility of starchy products with appropriate cooking instructions following a T2D diet for one week increased SDS content consumption in a real-life setting. The resulting increase in SDS intakes improved postprandial glycemic control in patients with T2D. In addition, the greater the SDS content, the greater the improvements in postprandial glycemic response parameters, as indicated by an inverse linear correlation between CGMS parameters and SDS content. This study opens new avenues for studying the beneficial effects of the high-SDS diet over a longer period of time to evaluate its impact on longer-term health benefits.

## Figures and Tables

**Table 1:** Slowly Digestible Starch contents of products selected for inclusion in the high-SDS and low-SDS diets, as measured at the start of the study.

Food Group	High-SDS Diet		Low-SDS Diet	
	SDS Range: Min–Max (g/100 g)	Number of Analyses	SDS Range: Min–Max (g/100 g)	Number of Analyses
Biscuits	21–25	34	NA	0
Rice	14–27	18	3–5	5
Pasta	13–22	21	2–12	14
Other wheat products	10–13	3	1–4	5
Bread/bread substitutes	2 *–12.5	7	0–4	6
Legumes	3–9	4	0–2	6
Other cereal products	NA	0	0–5	4
Potatoes	1 *	4	0–1	5

NA: Not Applicable for the diet. SDS = Slowly Digestible Starch. *: No high-SDS foods were identified; foods selected were low GI with controlled consumption frequency.

**Table 2:** Mean dietary intake during the one-week intervention for the high-SDS and low-SDS diets.

Dietary Intake	High-SDS Diet	Low-SDS Diet	*p* Value
Total daily energy intake (kcal)	1647 ± 58	1518 ± 68	<0.05
Available carbohydrates (g)	203 ± 9	178 ± 8	<0.001
Available carbohydrates (% of TDEI)	49 ± 1	47 ± 2	NS
Of Which SDS (g)	62 ± 5	12 ± 1	<0.0001
Of Which SDS (% of TDEI)	15 ± 1	3 ± 0	<0.0001
Proteins (g)	72 ± 4	70 ± 4	NS
Proteins (% of TDEI)	17 ± 1	19 ± 1	NS
Lipids (g)	56 ± 4	53 ± 6	NS
Lipids (% of TDEI)	30 ± 2	31 ± 3	NS
Dietary fibers (g)	24 ± 1	20 ± 1	<0.01
Dietary fibers (% of TDEI)	2.9 ± 0.1	2.6 ± 0.1	<0.05

Data are reported as mean ± SEM. SDS = Slowly Digestible Starch. TDEI = total daily energy intake. NS = not significant. *n* = 8 patients.

**Table 3:** Mean Slowly Digestible Starch intake during the one-week intervention for the High-SDS and low-SDS diets.

SDS Intake	High-SDS Diet		Low-SDS Diet		*p* Value *
	SDS (g/Day)	SDS/CHO (%)	SDS (g/Day)	SDS/CHO (%)	
SDS (g)/day	62 ± 5	30 ± 1	12 ± 1	6 ± 0.3	<0.0001
SDS (g)/breakfast	12 ± 2	27 ± 2	1 ± 0.3	3 ± 1	<0.0001
SDS (g)/lunch	23 ± 2	30 ± 2	8 ± 0.8	10 ± 1	<0.0001
SDS (g)/dinner	26 ± 3	32 ± 1	3 ± 0.3	4 ± 0.3	<0.0001

Data are reported as mean ± SEM. SDS = Slowly Digestible Starch. CHO = carbohydrates. *n* = 8 patients. * *p*-value for both the SDS and SDS/CHO data.

**Table 4:** Slowly digestible starch and carbohydrate intakes and contributions of the various food groups included in the high-SDS and low-SDS diets.

Food Group	High-SDS Diet (%)				Low-SDS Diet (%)			
	SDS Intake (g/6 Days)	Contribution to SDS Intake (%)	CHO Intake (g/6 Days)	Contribution to CHO Intake (%)	SDS Intake (g/6 Days)	Contribution to SDS Intake (%)	CHO Intake (g/6 Days)	Contribution to CHO Intake (%)
Biscuits	69 ± 9	18.7 %	204 ± 28	22.1 %	NA	NA	NA	NA
Rice	79 ± 10	21.5 %	159 ± 20	17.3 %	13 ± 2	18.7 %	89 ± 11	8.6 %
Pasta	152 ± 15	41.3 %	250 ± 25	27.1 %	26 ± 2	38.5 %	124 ± 11	16.0 %
Other wheat products	17 ± 2	4.6 %	27 ± 5	2.9 %	7 ± 1	10.4 %	67 ± 9	8.6 %
Bread/bread substitutes	22 ± 3	6.1 %	171 ± 22	18.5 %	13 ± 2	18.8 %	327 ± 28	42.2 %
Legumes	7 ± 1	1.9 %	19 ± 3	2.1 %	4 ± 1	5.9 %	44 ± 4	5.7 %
Other cereal products	NA	NA	NA	NA	5 ± 2	7.7 %	44 ± 9	5.7 %
Potatoes	22 ± 2	5.9 %	47 ± 5	5.1 %	0	0 %	34 ± 4	4.4 %
Other products containing CHO ^1^	0	0%	46 ± 9	5.0 %	0	0%	47 ± 9	6.1 %

Data are reported as mean ± SEM. NA = Not Applicable to the diet. SDS = Slowly Digestible Starch. CHO = carbohydrates. *n* = 8 patients. ^1^ Other products containing CHO include fruits, vegetables, dairy products, dessert, meat/fish/eggs, seasoning, sugar, and beverages.

**Table 5:** Parameter estimates and *P*-values from the linear model correlations between the SDS/carbohydrate content of meals and various postprandial glycemic responses.

	Estimate		*p*-Value
Parameter	Intercept	SDS/CHO	SDS/CHO
**Analysis of all three postprandial periods (*n* = 238)**			
tAUC 0–120 min	19,272	−4694	0.0006
Peak value	193	−55.4	<0.0001
Delta peak	71.3	−48.3	<0.0001
Time to reach the peak	99.5	8.34	0.6952
Time in Range 0–120 min			
<70 mg/dL	−0.009	0.14	0.2212
[70–140 mg/dL]	9.90	5.28	0.1287
[140–180 mg/dL]	8.12	3.71	0.2077
[180–250 mg/dL]	5.70	−5.86	0.0141
>250 mg/dL ^1^	1.27	−3.21	0.0043
**Analysis of lunch and dinner postprandial periods (*n* = 168) ^2^**			
tAUC 0–240 min	35,310	−2988	0.2043
Time in Range 0–240 min			
< 70 mg/dL	−0.012	0.19	0.2554
[70–140 mg/dL]	26.9	−6.10	0.3472
[140–180 mg/dL]	12.6	18.3	0.0031
[180–250 mg/dL]	8.35	−8.41	0.0546
> 250 mg/dL	1.13	−3.31	0.0625

SDS = Slowly Digestible Starch. CHO = carbohydrates. tAUC = total Area Under the Curve. ^1^ For this analysis, a quadratic model was found to be more appropriate (parameters described in Table 6). ^2^ The breakfast postprandial period usually did not last 4 h.

**Table 6:** Parameter estimates and *P*-values from the quadratic model correlations between the SDS/carbohydrate content of meals and various postprandial glycemic responses.

	Estimate			*p*-Value	
Parameter	Intercept	SDS/CHO	(SDS/CHO)^2^	SDS/CHO	(SDS/CHO)^2^
**Analysis of all three postprandial periods (*n* = 238)**					
Time in Range 0–120 min					
>250 mg/dL	1.06	−4.54	21.76	0.0003	0.0177

SDS = Slowly Digestible Starch. CHO = carbohydrates.

## Supplementary Materials

The following are available online at https://www.mdpi.com/2072-6643/12/8/2404/s1, Table S1: High-SDS diet composition, Table S2: Low-SDS diet composition.
